# Chilaiditi sign

**DOI:** 10.4103/0970-2113.68311

**Published:** 2010

**Authors:** Vipul D. Yagnik

**Affiliations:** *Consultant Endoscopic and laparoscopic surgeon, Ronak endo-laparoscopy and general surgical hospital, Patan-384265, Gujarat, India. E-mail: vipul.yagnik@gmail.com*

**Figure 1:**
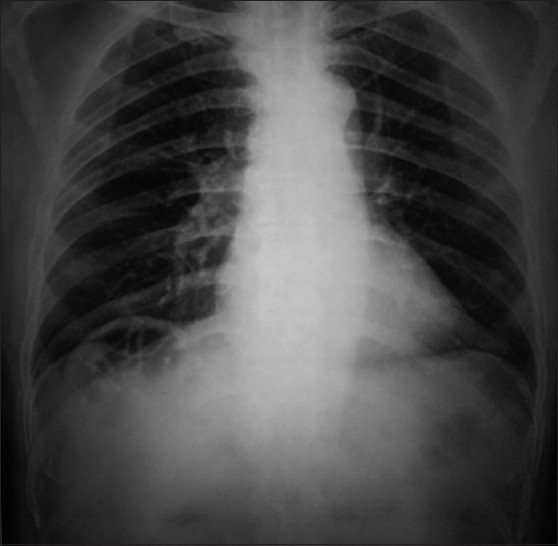
Chilaiditi sign.

Sir,

A 63-year-old male came for routine health check up. He was asymptomatic and had no significant past surgical or medical history. All routine blood investigations, pulmonary function test, ECG, 2D-echocardiography were normal. On examination, abdomen was soft and did not reveal any sign of peritonitis. His vitals were normal. Chest X-ray (PA View) revealed air under dome of right diaphragm [Figure 1].

Transposition of loop of colon in between diaphragm and liver is known as chilaiditi sign.[1] Incidence is around 0.1-1%. Most patients are asymptomatic.[1] Shortness of breath is a common manifestation of this condition. Sometimes, other symptoms like pain in abdomen, nausea and distention can be the presenting symptoms. Symptomatic presentation with chilaiditi sign is termed as chilaiditi syndrome. It was first described by a Greek radiologist Demetrius Chilaiditi in 1910. Chilaiditi sign may be seen in cirrhosis and COPD. Redundant mobile colon due to laxity of suspensory ligament of large intestine or liver may be the contributory cause of chilaiditi sign. Other conditions like colonic volvulus[2,3] and few colonic malignancies may be associated with chilaiditi syndrome. Diagnosis is usually made by X-ray; CT scan will help in confirmation of diagnosis in case of suspicion. Most patients respond to medical management, and surgery is reserved for those who do not respond to usual conservative line of management. This condition is important for chest physicians because few patients with breathlessness may present with this type of X-ray picture and respiratory pathology is not needed in such cases. All chest physicians should be aware that shortness of breath is not always due to problem in the chest.
